# Detecting non-neutral modules in species co-occurrence data: principles and application to plant communities

**DOI:** 10.1098/rsos.241375

**Published:** 2025-07-16

**Authors:** Fabien Laroche, Bodil Ehlers

**Affiliations:** ^1^ UMR DYNAFOR, Université de Toulouse, INRAE, Castanet-Tolosan 31326, France; ^2^ Department of Ecoscience, Aarhus Universitet, Aarhus C. 8000, Denmark

**Keywords:** biodiversity, network, clustering, niche, metacommunity, jump process

## Abstract

Inferring assembly processes from species co-occurrence data is a long-standing challenge in community ecology. Approaches that focus on detecting non-random spatial covariance between species occurrences are limited by the fact that spatial patterns can deviate from randomness for many reasons. Process-based null hypotheses are needed to overcome this limitation. Here, we explored the neutral theory of community ecology as a promising candidate. We built upon a robust property of neutral co-occurrences, the ‘rank consistency’: within a common regional pool, the presence probabilities of two species should be ordered similarly across local sites. We suggested performing pairwise tests of species rank consistency along ecological gradients of interest and creating a species network where rank-consistent species are connected. Network mo­dules then indicate species groups that do not co-occur neutrally with one another, hence making an important step towards the understanding of assembly processes. These modules can be further interpreted by relating their composition to species traits. We tested our framework on virtual data and successfully retrieved pre-defined functional groups without generating false positive detections. Then, we analysed two published examples on tropical trees and Mediterranean herbaceous communities. We found ecologically meaningful modules in both cases, hence illustrating the potential of our approach.

## 1. Introduction

Understanding the mechanisms driving the coexistence of species is a central question of community ecology. Spatio-temporal analysis of species co-occurrences (i.e. their simultaneous presence within the same sampling units) is a classic approach to that question [1]. However, whether species co-occurrences can really inform on coexistence mechanisms is debated [2]. Coexistence mechanisms do not always have straightforward implications on co-occurrences, like competitive interactions among more than two species or asymmetric interactions [3]. Moreover, the same co-occurrence pattern may result from many mechanisms: spatial segregation of species can stem from distinct environmental niches or competitive exclusion within the same environmental niche. These ambiguities call for rigorous methods to avoid misinterpretation.

The neutral theory [4,5] provides a robust methodology for studying species community patterns, including co-occurrences. It assumes that all individuals are ecologically equivalent: when placed in the same local community, they have identical fitness and identical impact on other individuals’ fitness, irrespective of the species they belong to. Their vital rates may vary with the environmental context, but always in the same way for all individuals and species. Community patterns are thus solely driven by the stochasticity of birth, death and dispersal events affecting individuals, and by global changes among communities in migration rate and local fitness, which affect all individuals in the same way. The neutral theory thus provides a null hypothesis to detect the effect of fitness differences (e.g. ecological niches) shaping the assembly of communities [6,7].

Classic approaches of co-occurrence data are based on comparing observed patterns with randomized species-per-sample contingency tables (i.e. ‘null’ models [8]). Finding patterns that cannot also be generated by the neutral theory with such approaches has been surprisingly challenging. For instance, the C-score, a community-wide statistic, which quantifies how much species occurrences tend to covary negatively in space [9], shows similar deviations from randomized contingency tables in empirical systems [10,11] and in neutral simulations [12]. This stems from the fact that a purely neutral dynamics can generate strong negative covariance of occurrences among species when the competition among individuals is symmetrical but very intense within communities, but can also generate strong positive covariance among species occurrences when some ecological gradient induces marked variations in the fitness or the immigration rate of individuals among communities. The C-score and other community-wide statistics based on the average value of the covariance between species occurrences among communities may thus not be adapted to detect deviation from neutral theory, especially when compared to randomized expectations.

Community-wide statistics on species co-occurrences use limited information about presence–absence data. In particular, they overlook species identities, their functional traits and phylogenetic relatedness. Bell [12] and Bell *et al*. [13] suggested that separately analysing distinct phylogenetic or functional groups of species may yield more frequent positive co-occurrences within groups than those observed at the whole community level, which could be interpreted as deviating from neutrality. Although confirmed by empirical observations, their conjecture lacked a clear theoretical justification.

The idea of considering patterns within and among well-chosen species groups has been more developed for neutrality tests based on species relative abundances within communities, on both temporal [14] and spatial [15] patterns. In these tests, inconsistencies between patterns of relative abundances at groups and individual species levels indicate that species do not behave neutrally within groups or that groups interact in a non-neutral way with one another (while species may still be neutral within groups). The second interpretation is reminiscent of the idea that communities can harbour functional redundancy within well-separated functional groups, as evidenced in empirical studies [15,16], potentially creating ‘guilds’, i.e. groups of species exploiting the same array of resources in a similar way [17]. We were not aware of analogous work on co-occurrence data.

Neutrality tests based on species relative abundances are more advanced than those based on co-occurrence data on a second aspect. Empirical studies trying to reject neutrality often consider one particular neutral model, which comes along with instrumental hypotheses potentially obscuring the interpretation of test outcomes [3]. A considerable effort has been devoted to relaxing instrumental assumptions of neutral models predicting species relative abundances, like the zero-sum assumption [18,19]. By contrast, very little has been done for neutral co-occurrence patterns.

Here, we aimed to develop a framework to infer the presence of groups of species with non-neutral interactions among groups from co-occurrence data. We used a robust property of the spatially implicit neutral theory that does not require additional instrumental hypotheses: the ‘rank consistency’ of species.

## 2. Material and methods

### 2.1. Rank consistency in spatially implicit neutral models

We considered a spatially implicit neutral model [5,20] where J species are present in an infinite regional pool with constant relative abundances $\pi_{1},\pi_{2},...,\pi_{J}\in \left[0,1\right]$. We assumed that n local sampling units are connected to the regional pool through limited dispersal. Neutral theory implies that differences in the probability of the presence of species within the local sampling unit i can only come from differences in their relative abundance in the regional pool. If two species have equal regional relative abundances, their probabilities of presence are identical within any sampling unit i. If a species has a higher regional relative abundance than another, it has a higher local probability of presence in any sampling unit i. Focusing on two species with arbitrary labels 1 and 2, this basic property can be formally expressed as


(2.1) $$\pi_{1}\leq \pi_{2}\;⟹\;p_{1}^{\left(i\right)}\leq p_{2}^{\left(i\right)}\text{ for any sampling unit }i,$$

where $p_{1}^{\left(i\right)},p_{2}^{\left(i\right)}$ are the presence probabilities of species 1 and 2 in sampling unit i, respectively. We call property (2.1) the ‘rank consistency’ of species 1 and 2 across sampling units.

### 2.2. Testing rank consistency of two species

The rank consistency between species 1 and 2 relies on the presence probabilities of species in sampling units (see equation (2.1)), not on realized presences. Assessing it in co-occurrence data thus calls for a statistical test. Here, we explored a robust non-parametric test (see Discussion for alternative parametric strategies). We selected sampling units where one and only one species of the pair {1,2} is present (assuming that there is more than one such sampling unit). These sampling units are divided into two non-empty, disjoint subsets, namely $A^{*}$ and $B^{*}$. Rank consistency implies that observing species 1 in more than half of the sampling units in $A^{*}$ and less than half of the sampling units in $B^{*}$ (or the reverse configuration) is unlikely. Denoting $N_{1}^{A^{*}}$ and $N_{1}^{B^{*}}$ as the number of sampling units occupied by species 1 in $A^{*}$ and $B^{*}$, respectively, this idea leads to a test of rank consistency for a species pair based on the bivariate statistics $\left(N_{1}^{A^{*}},N_{1}^{B^{*}}\right)$ (see electronic supplementary material, appendix S1, for details). We implemented the test using R software [21]. All the code and information needed to reproduce the analyses presented below are provided on a public online repository [22].

### 2.3. Testing along ecological gradients

The output of the rank consistency test depends on how sampling units are divided into $A^{*}$ and $B^{*}$. For instance, a random split would lead to a similar proportion of sampling units where species 1 is present in each subset. The rank consistency would likely not be rejected with such a strategy, irrespective of the biological context.

**Figure 1.**
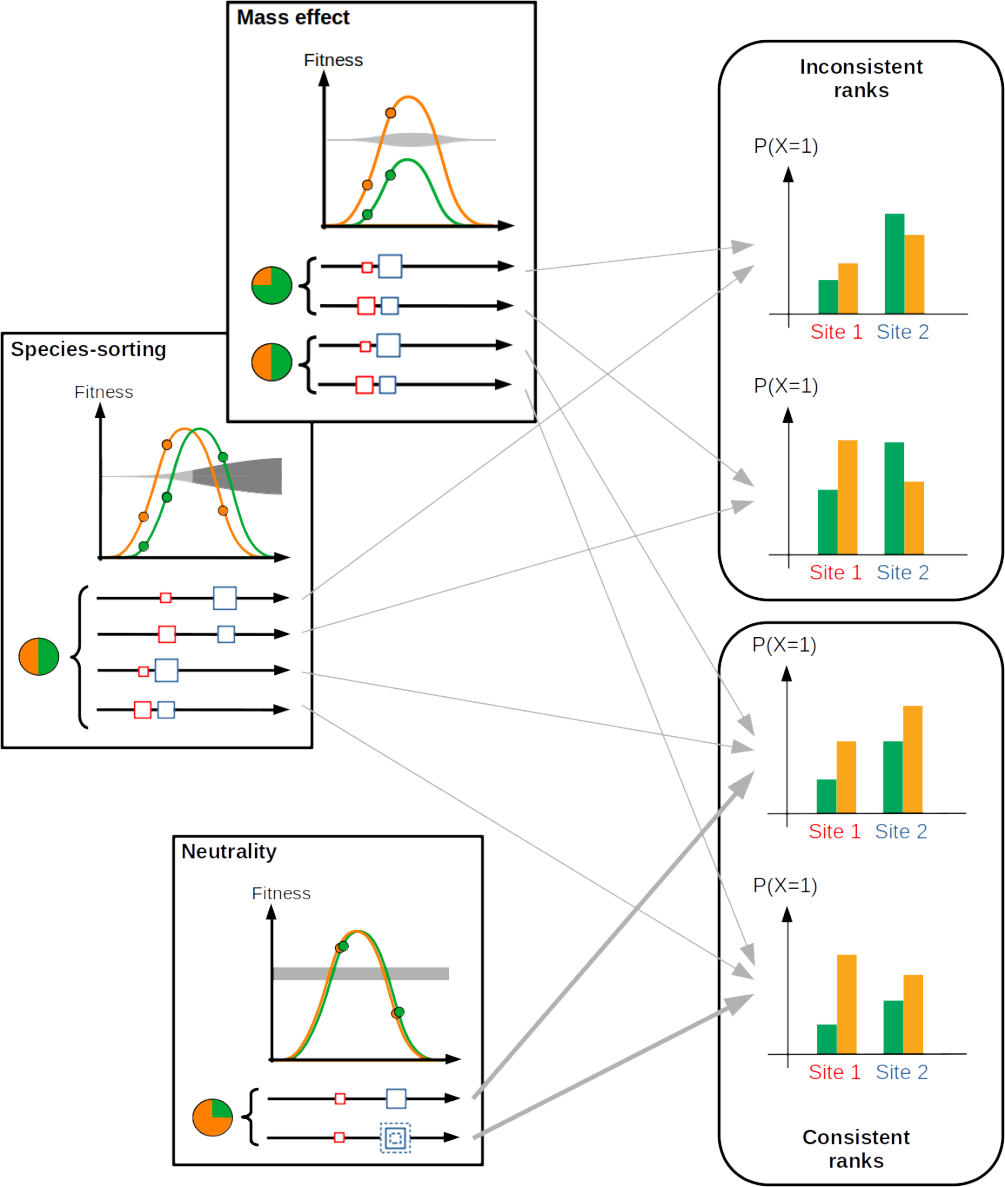
From ecological scenarios to the rank consistency of a species pair. Three scenarios are presented in the left panels. In each scenario, the green and orange curves show the fitness of the two species along an ecological gradient. The grey shape in the background denotes the fitness ratio of green over orange species (darkest grey indicates values above 1). Various positions of a pair of sampling units are presented with squares along replicated *x*-axes (and reported as circles on species fitness curves). The square size indicates the community’s carrying capacity. In the neutral scenario, the carrying capacity in sampling unit 2 can fluctuate in time (several superimposed squares with dashed lines; see electronic supplementary material, appendix S2, for an example). A pie chart shows the relative abundances of species in the regional pool associated with each example of sampling unit configuration. Bar plots show possible configurations of presence probabilities of the two species in the two sampling units. Grey arrows indicate when a configuration within a scenario can reach one type of bar plot. Rank inconsistency cannot be obtained with neutral scenarios.

Choosing subsets of sampling units $A^{*}$ and $B^{*}$ that discriminate distinct conditions along an ecological gra­dient of interest can be more informative. By ecological gra­dient, we meant a variation in some abiotic or biotic feature that is thought to influence the community of interest and has been measured independently from community observation. It can relate to local climatic conditions, resource availability, the presence of a predator or a facilitator, etc. Rejecting rank consistency in this case means that species 1 occurs more often than species 2 at one end of the gradient, while the reverse is true at the other end. This can only happen if the fitness ratio of species 2 over species 1 increases from the former to the latter situation, hence violating the neutrality assumption (figure 1). The fitness ratio can increase for various reasons: both species’ fitness may increase along the gradient but faster for species 2 than for species 1 (in figure 1, any sampling unit configuration under ‘mass effect’, third and fourth sampling unit configuration under ‘species sorting’), or fitness of species 1 may decrease while fitness of species 2 increases (in figure 1, first and second sampling unit configuration under ‘species sorting’, emblematic of the species-sorting effect [23]), or both species’ fitness may decrease but faster for species 1 than for species 2.

Rejecting rank consistency is different information from sharing or not sharing the same variation of presence probability along an ecological gradient. Species can harbour similar variations of their presence probability along the gradient but violate rank consistency. However, they can also show a contrasted response to a gradient and still be rank-consistent (see the four-bar plots in figure 1).

At last, while neutrality cannot generate rank inconsistencies—the central property we built upon here—the reciprocal is not true: non-neutral dynamics like ‘species sorting’ or ‘mass effect’ (as defined by Leibold *et al*. [23] and illustrated in figure 1) can generate rank-consistent species pairs. Therefore, only rejecting rank-consistency tests carries information, not accepting it.

### 2.4. Module detection and interpretation

The test developed in the previous section can determine whether one pair of species deviates from rank consistency, hence violating the neutral assumption. Iterating the test across the J(J−1)/2 species pairs in the community yields a symmetric species-by-species matrix of *p*-values. One can view this matrix as the adjacency matrix of a species network, where species pairs are connected if the rank-consistency test is not rejected at some target level (here, we take p≥0.05). Moving to a network perspective offers the opportunity to look for modules in the graph, i.e. subsets of densely connected species. Modules correspond here to neutral species clusters, groups of species that co-occur neutrally within the group but not neutrally with species outside the group. We used methods for module detection provided in the R interface of *igraph* software [24].

One expects species belonging to distinct modules to harbour functional differences explaining their deviation from neutrality, like a trait driving the variation of their fitness ratio along an ecological gradient (see figure 1). Searching for traits of species that drive their attribution to one module or another can thus foster the biological understanding of modules. To do so, we used a classification tree based on the CART algorithm implemented in the R package *rpart* [25]. The algorithm builds a decision tree that attributes a module to each species based on its traits, with quantified classification error. Classification trees were pruned to keep only the splits that generated a significant drop in the classification error (evaluated by cross-validation).

### 2.5. Environmental filtering model

We used an environmental filtering model adapted from [15] to test the framework introduced above. Similarly to our neutral framework, it is a spatially implicit model of metacommunity, where local sampling units receive immigrants from a regional pool with constant relative abundances. The model is a multivariate jump process tracing the abundance of each species in time. The birth, death and immigration rates of species depend on the distance between a species trait and an optimal value for this trait within sampling units. Denoting $t_{j}$ as the trait value of species j, and $t_{\text{opt}}^{\left(i\right)}$ the optimal trait value to live in a sampling unit i, one defines a fitness coefficient of species j in unit i:


$$w_{ij}=\exp\left(-{\left(t_{j}-t_{\text{opt}}^{\left(i\right)}\right)}^{2}\right)$$

which decreases as the species trait departs from the local optimal value. The *per capita* birth rate, *per capita* death rate and immigration rate of species j in sampling unit i are, respectively, set equal to


$$\begin{array}{l} b_{ij}=w_{ij}^{\Omega_{b}}\;b_{i}\left(\sum_{j=1}^{J}A_{ij}\right),\\ d_{ij}=w_{ij}^{-\Omega_{d}}\;d_{i}\left(\sum_{j=1}^{J}A_{ij}\right),\\ M_{ij}=w_{ij}^{\Omega_{m}}I_{i}\pi_{j}\times b_{ij}, \end{array}$$

where $\Omega_{b},\Omega_{d},\Omega_{m}$ denote the selection intensity on rates induced by environmental filtering on the species trait, $A_{ij}$ is the local abundance of species j in sampling unit i, and $b_{i},d_{i}$ are functions that only depend on the total number of individuals in the community (a ‘community-level density dependence’ [19]) and allow a stationary distribution of local species abundances to emerge. As a result, a species j with a trait $t_{j}$ further from the local optimum $t_{\text{opt}}^{\left(i\right)}$ had a lower local *per capita* birth rate and immigration rate while its *per capita* death rate was higher.

Assuming that the community composition has reached a stationary distribution in sampling unit i, we used previously published results [15,26,27], which showed that the composition of a community with a individuals can be simulated following three steps. First, one samples a vector of local species relative abundances $\left(g_{1},...,g_{J}\right)$ in a Dirichlet distribution with parameters $I_{i}w_{ij}^{\Omega_{m}}\pi_{j}$ for 1≤j≤J and accepts the proposed sample with probability


$$P_{\text{accept}}={\left(\frac{\sum_{j=1}^{J}g_{j}w_{ij}^{\Omega_{b}+\Omega_{d}}}{\max_{1\leq l\leq J}w_{il}^{\Omega_{b}+\Omega_{d}}}\right)}^{a}.$$

Sampling is repeated until acceptance. Second, one computes transformed local relative abundances:


$${\tilde{g}}_{j}=\frac{g_{j}w_{ij}^{\Omega_{b}+\Omega_{d}}}{\sum_{l=1}^{J}g_{l}w_{il}^{\Omega_{b}+\Omega_{d}}}.$$

Third, one samples the species labels of the a individuals of the community using a multinomial distribution with size a and probabilities $\left({\tilde{g}}_{1},...,{\tilde{g}}_{J}\right)$.

When all the $w_{ij}$ are constant (i.e. all the species have the same trait value), or when $\Omega_{b}=\Omega_{d}=\Omega_{m}=0$ (i.e. no selection applies on the trait), the procedure above takes a simpler form. The composition of a community with a individuals in sampling unit i is obtained by first sampling local species relative abundances $\left(g_{1},...,g_{J}\right)$ in a Dirichlet distribution with parameters proportional to $\pi_{j}$, automatically accepting the sample and then sampling species labels in a multinomial distribution with size a and probabilities $\left(g_{1},...,g_{J}\right)$. This yields a Dirichlet-multinomial distribution which corresponds to the prediction of spatially implicit neutral models [27,28]. The special non-neutral case, where $\Omega_{b}=\Omega_{d}=0$ and $\Omega_{m}>0$, was implemented in the *ecolottery* package of R software [29].

### 2.6. Virtual data

We used the environmental filtering model to generate virtual data and test our neutral module detection framework. We tested three values of numbers of sampling units n=50, 100, 200 to explore various levels of statistical power. Numbers of individuals (a) varied across sampling units and were sampled in a negative-binomial distribution with a mean of 100 and a coefficient of variation of 0.25. Immigration parameter (I) varied across sampling units and was sampled in a Γ-distribution with a mean of 5 and a coefficient of variation of 0.25 in each unit. We set the regional pool species richness at J=100 species. The vector of regional abundances of species $\left(\pi_{1},...,\pi_{J}\right)$ was sampled in a Dirichlet distribution on the simplex of dimension J, with the same parameter θ for all species. A higher θ yields a more even regional abundance distribution. We explored three values corresponding to markedly distinct levels of evenness: θ=0.04, 0.2, 1.

For each sampling unit i, we sampled an optimal trait value $t_{\text{opt}}^{\left(i\right)}$ in a uniform distribution between 0 and 1. The gradient of $t_{\text{opt}}^{\left(i\right)}$ across sampling units corresponds to the ecological gradient of interest in our virtual study. We considered scenarios with F=1, 2, 3 groups of species such that all the species within a group had the same trait value, and trait values differed among groups. A group thus corresponds to a set of species with identical ecological niches along the environmental gradient. When F=1, the trait of the single group was set to t=0.5. When F=2, the trait values of the two groups equated to 0.25 and 0.75, respectively. When F=3, the trait values of the three groups equated to 0, 0.5 and 1, respectively. We randomly attributed one group among the F options to each species j. We considered that filtering occurred on both *per capita* birth rate and immigration rate with similar intensity, i.e. $\Omega_{b}=\Omega_{m}=\Omega >0$ and $\Omega_{d}=0$. We tested three increasing levels of filtering intensity: Ω=0, 2.77, 11.98. As commented earlier, the case Ω=0 corresponds to the neutral assumption. A value of $\Omega_{b}=2.77$ (respectively $\Omega_{b}=11.98$) induces a drop of 50% (respectively 95%) in birth and death rates for a species well adapted to the middle of the environmental gradient when placed at one end of the gradient.

For each combination of number of sampling units n, number of groups F, level of filtering intensity Ω and regional pool evenness θ, we simulated 30 replicates of a presence–absence dataset. We thus generated a total of 3×3×3×3×30=2430 virtual contingency tables. We applied our neutral module detection framework to the 2430 datasets. We used traits $t_{j}$ as a species trait input to compute a classification tree of modules. Species that were present in less than five sampling units were removed before the analysis, for we know that they cannot lead to a rejection of a rank-consistency test (see rank-consistency test presentation in [22]).

### 2.7. Real plant community data

#### 2.7.1. Tropical tree communities of the Western Ghats, India

We considered a published dataset reporting the abundance of tropical trees in 971 ha plots spread over approximately 22 000 km^2^ along a gradient of annual rainfall in the Western Ghats, Karnataka state [30]. In each plot, all trees above 10 cm diameter at breast height were identified, which yielded a dataset of 57 090 trees, with 61–1883 individuals per plot. The complete dataset is available in Ecological Archives (http://esapubs.org/archive/ecol/E091/216/), with accession number E091-216. Here, we considered the simplified dataset provided in electronic supplementary material, data S1, of [15]. We considered the same two species traits as those authors. The first trait is the potential stratum position of species as documented by Ramesh *et al*. [30]: T5 (>34 m height), T4 (24–34 m), T3 (16–24 m), T2 (8–16 m) and T1 (<8 m). The second trait is the leaf shedding phenology: deciduous (‘D’) and evergreen (‘E’) species.

By comparing the relative abundance of species in plots with the relative abundance of deciduous and evergreen individuals, Laroche *et al*. [15] showed that the turnover from deciduous to evergreen communities was stronger than the neutral prediction. Communities with a higher proportion of deciduous indivi­duals occurred in plots with lower precipitation, in line with the fact that deciduousness is an adaptation to drought. Here, we assessed whether the same patterns could be obtained using the presence–absence of tree species only, analy­sing species modules obtained from pairwise tests of rank consistency based on the annual rainfall gradient (measured by Ramesh *et al*. [30]).

#### 2.7.2. Herbaceous communities in the Saint-Martin-de-Londres basin, France

Our second example is a vegetation survey of the Mediterranean garrigue in the Saint-Martin-de-Londres basin, France. The survey took place in 2010, over 12 sites of approximately 1 ha is dominated by wild thyme (*Thymus vulgaris* L.), an aromatic shrub. Within a site, 50 quadrats of size 1 m ×0.5 m were laid out, and all plant species inside were identified. Two types of quadrats were considered: 25 quadrats were chosen to contain at least one thyme plant, while the other 25 quadrats were chosen to ensure that thyme was absent from inside the quadrat and the nearest 1 m around it. For the quadrats without thyme, care was taken to avoid rocky, shallow soil, which could create bias in a number of species. This survey was analysed in a previous study by Ehlers *et al*. [31] and the dataset has been made available online [32].

Wild thyme produces monoterpenes as the main chemical compounds in its aromatic oil, and this species has a genetic polymorphism that produces different types of dominant monoterpenes [33]. Thyme monoterpenes have a direct allelopathic effect on other plants, which varies with the type of monoterpene produced [34–36]. Some plants may be more adapted to specific types of thyme monoterpenes than others [37–39]. The presence of thyme monoterpenes in the soil can alter competitive interactions among neighbouring plant species [40].

The present dataset was used by Ehlers *et al.* [31] to test the idea that thyme presence in a quadrat could increase species richness locally by reducing the abundance of a dominant competitor, the perennial grass *Bromus erectus*, through a direct allelopathic effect. The authors found a reduced abundance of *B. erectus* in the thyme quadrats where thyme produced carvacrol as a dominant monoterpene, and this decrease in abundance was correlated with an increase in the total species richness. In the same vein Ehlers & Thompson [37] had previously found, within the same study area, that *B. erectus* populations originating from sites where thyme produced a non-phenolic monoterpene showed local adaptation to non-phenolic thyme soil, whereas *B. erectus* populations originating from sites where thyme produced a phenolic monoterpene (carvacrol or thymol) did not show adaptation to phenolic thyme soil, suggesting that *B. erectus* could adapt to the presence of non-phenolic thyme but not to the pre­sence of phenolic thyme.

Here, we thus focused on the five sites (252 quadrats, one site had two additional quadrats with thyme) where thyme produced a phenolic monoterpene in the study [31], and we tested whether our approach could detect modules in plant communities when considering thyme presence as the ecological gradient of interest. We expected the analysis to reveal a module corresponding to competitors like *B. erectus*, which would be excluded close to thyme and another module corresponding to species that benefit from the subsequent competition release effect and become more present close to thyme. Two species traits were used in the analysis: life strategy (‘annual’ or ‘perennial’) and plant minimum and maximum heights. We also included the family taxonomic level of plants as complementary information. These features were manually compiled from [41].

## 3. Results

### 3.1. Virtual data

Detailed simulation outputs have been provided on a public online repository (see virtual study in [22]). The total species richness varied across the 2430 virtual datasets, from approximately 10 to 90 species. This reflected the variable outcomes of the two consecutive sampling processes that generate virtual datasets—the sampling of the regional species pool by the environmental filtering model and the *post hoc* filtering of species below five occurrences—depending on parameter values. The evenness of the pool (θ) had a strong positive effect on total species richness within datasets, explaining approximately 50% of the observed variance. The number of sites (n) positively affected the species richness and explained approximately 20% of the variance. Selection intensity (Ω) negatively affected the species richness and explained approximately 5% of the variance. The number of groups F had a small negative effect on the species richness, explaining approximately 1% of the variance (see virtual study in [22] for more details).

**Figure 2.**
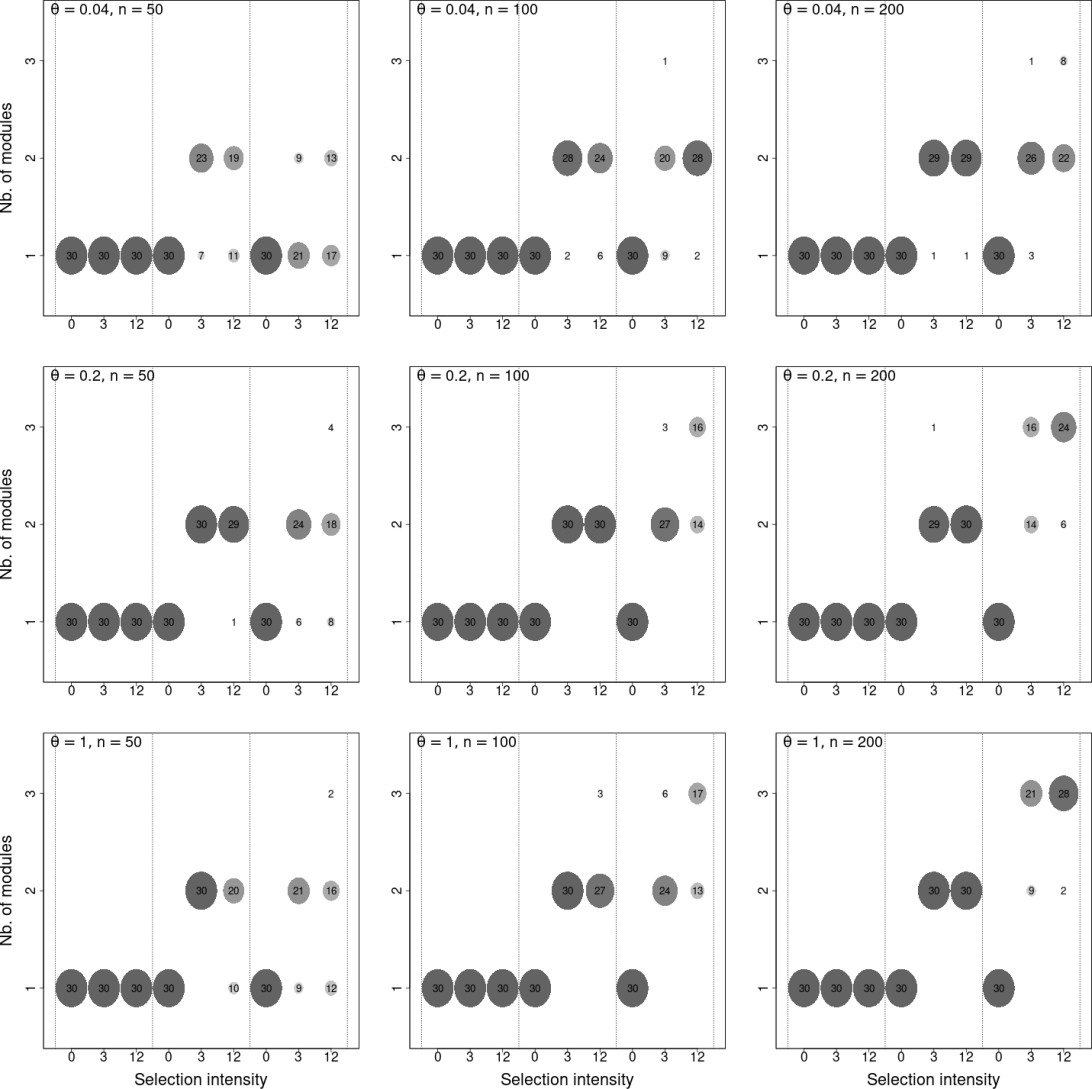
Number of modules detected in virtual datasets with various numbers of sampling units, regional pool evenness values, number of functional groups in the pool and selection intensity values. Panels correspond to combinations of a number of sampling units (n=50, 100, 200; in columns) and a regional pool evenness (θ=0.04, 0.2, 1; in rows). Each panel is divided into three parts (delineated with dashed lines) corresponding to simulations with F=1, 2, 3 functional groups, respectively. In each part, the distribution of the number of modules (vertical axis) detected over the 30 replicates is reported for each selection intensity value (horizontal axis; Ω). The number of replicates for which a given number of modules has been detected is reported as a disc, the radius and darkness of which are proportional to the number of replicates involved. The number of replicates is reported within the disc.

In the 1350 simulated datasets, where the neutral assumption was verified (i.e. when F=1 or Ω=0), only one single large module was detected (figure 2), suggesting that our framework did not generate false-positive detection of modules. This result was robust to changing the module detection method.

Our benchmark of module detection methods (see virtual study details in [22]) indicated that for all the other aspects of our analyses (power of module detection, number of detected modules), the Leiden algorithm [42] visually showed the best results. Therefore, we present below the analyses obtained with this algorithm.

Among the 540 non-neutral datasets with F=2 functional groups and Ω>0, we correctly detected two modules 497 times (92% of success). In 39 datasets, only one module was detected. This occurred when the number of sampling units was low (n=50) or when the regional pool was highly uneven (θ=0.04; figure 2), which corresponded to low species richness values among simulated datasets. Three modules were very rarely detected (four datasets only).

Among the 540 datasets with F=3 functional groups and Ω>0, we detected one module in 87 datasets, two modules in 306 datasets and three modules in 147 datasets. Non-neutrality (two modules or more) was thus detected in 453 datasets (84%). The failure to detect non-neutrality occurred mostly when the number of sampling units was low (n=50) and the regional pool was highly uneven (θ=0.04), which corresponded to the lower end of the range of species richness explored in our virtual study (approx. 10 species). The correct number of modules was found only in 147 datasets (27%). This suggested that detecting more than two modules along the ecological gradient was more challenging. Detecting two modules instead of three occurred mostly when the number of sampling units was low (n=50) or when the regional pool was highly uneven (θ=0.04). In more intermediate situations (n≥100 and θ≥0.2), module detection was more accurate (figure 2). Overall, a larger number of sampling units and a more even regional pool—two factors that increase species richness in the dataset—both improved the power to detect non-neutrality and the accuracy in the number of modules detected.

We further analysed whether the species trait $t_{j}$ significantly explained the composition of the modules derived above. Among the 497 datasets where F=2 functional groups were simulated and two modules were detected, we obtained that a classification tree based on the species trait significantly contributed to discriminating the composition of modules in 85% of the cases (421 datasets). Among the 147 datasets where F=3 functional groups were simulated and three modules were detected, we obtained that a classification tree based on the species trait significantly contributed to discriminating the composition of modules in 69% of the cases (102 datasets), while it could only discriminate one module from the two others in 24% of the cases (36 datasets).

### 3.2. Tropical tree communities of the Western Ghats

We considered the 176 species that occurred in at least five plots among the 96 plots of the Karnataka dataset. We ran pairwise rank-consistency tests, splitting plots according to the rainfall gradient. We obtained the rank-consistency matrix upon which we could apply the Leiden algorithm of module detection. The Leiden algorithm is stochastic, and its output can vary from one run to another. We, therefore, ran it 500 times and identified species that occur in the same modules across replicates, hence defining robust cores of modules (see Karnataka study details in [22]).

**Figure 3.**
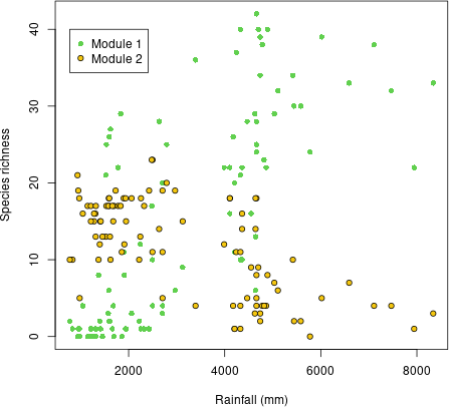
Number of tree species from each module in Karnataka plots along the annual rainfall gradient. For each plot, two dots are reported on the graph at the annual rainfall value corresponding to the plot: a green dot shows the number of species from module 1 (mostly evergreen species), a yellow dot shows the number of species from module 2 (most of the deciduous species and some evergreen species).

We detected two modules in the network of pairwise rank-consistency tests, hereafter called modules 1 and 2. The robust cores of modules 1 and 2 contained 51 and 35 species, respectively (the remaining 90 species were not consistently attributed to modules across replicates). These modules were clearly segregated along the rainfall gradient, with module 1 being more present at the higher end, in wet plots, while module 2 was more present at the lower end, in dry plots (figure 3).

The classification of species among module cores based on species traits revealed that leaf shedding phenology was a strongly significant factor involved in the segregation among modules. While evergreen species occurred in both modules, although more frequently in module 1 (50 species in module 1, 12 species in module 2), deciduous species nearly all occurred in module 2 (23 species) with only one exception. Therefore, as mean annual rainfall increases, the fitness of a functional group associated with most deciduous species and some evergreen species declines relative to the fitness of a functional group associated with mostly evergreen species.

### 3.3. Herbaceous communities in the Saint-Martin-de-Londres basin

We considered the 82 species that occurred in at least five quadrats among the 252 quadrats of the dataset. Four quadrats were removed, for they contained less than two species. The working dataset thus resulted in the occurrences of 82 plant species in 248 quadrats (121 quadrats without thyme and 127 quadrats with thyme). We ran pairwise rank-consistency tests splitting quadrats according to the presence or absence of thyme. We obtained the rank-consistency matrix upon which we could apply the Leiden algorithm of module detection, here again replicating the analysis 500 times to obtain robust cores of modules.

We detected two modules in the network of pairwise rank-consistency tests, hereafter called modules 1 and 2. The robust cores of modules 1 and 2 contained 11 and 8 species, respectively (the remaining 63 species were not consistently attributed to modules across replicates).

**Figure 4.**
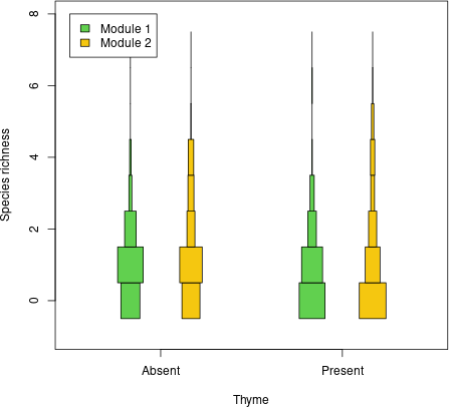
Distribution of the number of species from each module in quadrats without and with thyme. Distribution of species richness is shown using violin plots, i.e. vertical histograms. For each module (colour) and each modality of thyme occurrence (presence/absence), horizontal bars show the proportion of quadrats where a given species richness (*y*-axis) is observed.

Species richness of modules showed a weak drop between no-thyme and thyme quadrats (figure 4; an average drop of 12% and 8% in thyme quadrats for modules 1 and 2, respectively). None of these trends was significant (effective *p*-value in a binomial generalized linear model equated to 0.29 and 0.40 for modules 1 and 2, respectively). Although not significant, there seemed to be a more pronounced average drop in species richness for module 1, which may have induced the switch of ranks among modules when moving from no-thyme to thyme quadrats.

The classification of species among module cores based on species traits (plant height, life strategy and taxonomic family) revealed no effect of traits but a clear segregation of taxonomic families among modules: module 2 contained Poaceae (including *Bromus hordeaceus*), Lamiaceae and Fabaceae species, while module 1 contained distinct families (Apiaceae, Asparagaceae, Asteraceae, Buxaceae, Caryophyllaceae, Geraniaceae, Rosaceae), except one Fabaceae species.

## 4. Discussion

We built a framework to search for groups of species with non-neutral interactions among groups based on co-occurrence data. We started from the robust neutral prediction that, in a spatially implicit design, the presence probabilities of two species should be ordered in the same way in all sampling units: the ‘rank-consistency’ property. We showed how to test the rank consistency of two species along an ecological gradient of interest based on their co-occurrence pattern. We suggested compiling the outcomes of pairwise tests of species pairs into a network where only rank-consistent species are connected and searching for modules in this network. By definition, species tend to be rank-consistent within modules but not among modules. Modules thus identify species groups with non-neutral interactions among groups: the fitness ratio of two species belonging to distinct modules varies along the ecological gradient of interest. We suggested using species traits to biologically interpret these modules. We tested our approach on simulated datasets before applying it to two published datasets of plant communities.

Many studies have built species networks from species co-occurrence data, aiming at generating insights on species interactions (with acknowledged limitations [43]). Recent examples made use of Markov networks [44] or Gaussian graphical models [45] to estimate spatial associations among species while controlling for the composition of the rest of the community. Some of these studies have focused on interpreting links and modules in these networks in terms of ecological gradient [46] or species traits and phylogeny [47]. However, our approach differs from these frameworks in the essential feature that, in the species network considered here, links do not quantify whether species occurrences covary in space but whether species have consistent ranks along an ecological gradient, which is a totally distinct property (figure 1). The main advantage of taking this new perspective is that, contrary to deviation from the random spatial association, deviation from rank consistency has a clear meaning in terms of interactions between two species: it indicates the existence of non-neutral interactions (i.e. a variation in fitness ratios between species). Our approach thus makes a new step towards the understanding of community assembly processes from co-occurrence patterns because it is able to discard a process-based null hypothesis, the neutral theory and the point where non-neutral processes are necessarily at work. By contrast, because our approach is made to reject neutrality, not to accept it, belonging to the same module should not be interpreted as a positive result of neutral dynamics but simply as an absence of power to detect deviation from neutrality based on the rank-consistency test.

In our spatially implicit framework, there are only two scales: (i) the local sampling unit, where space does not play any role, and which therefore corres­ponds to a scale where all individuals can interact with one another on a reasonably short time scale and (ii) the regional scale which is shared by all sampling units as a common migrant pool. This framework does not allow considering larger sampling units with internal spatial structure nor accounting for hierar­chical spatial structure in the sampling design (e.g. quadrats within site in our study of herbaceous communities). Both limitations are harmless in our real case studies: sampling units are small (quadrats of <1 m^2^ for herbs and plots of 1 ha for trees), and the study on herbaceous communities crossed the ecological gradient and site identity in a balanced design. Including more scale in our framework is a fascinating perspective, though, which could, for instance, build from multi-level spatially implicit models like the one proposed by Munoz *et al.* [48].

The virtual experiment showed that our approach detected modules without false-positive detection and with sufficient power for realistic ecological applications (figure 2). Studies with 100 sampling units or more and moderate-to-high species diversity (ty­pically 20 species or more in sampled communities; see virtual study details in [22]) are particularly favourable for module detection. Interes­tingly, while the presence of modules seemed easy to detect, finding more than two modules seemed feasible but more demanding in our virtual study. The functional resolution of our approach may be improved in several ways. A simple option could be to iterate the method on the subset of species belonging to one module and check whether it can be further split. However, this would come with a drop in species richness, which we saw could degrade power. Other options would revolve around increasing the power of the rank-consistency test. The test we built is distribution-free and conservative, which maximizes robustness but necessarily comes at the cost of power. Tackling rank consistency through the prism of joint species distribution models (JSDM) [49] is a stimulating perspective: JSDM could be used to predict the presence probability of species within sampling units with confidence intervals and *post hoc* testing of rank consistency on these predictions could then be applied. We see here a promising avenue to improve the power to detect modules and account for more than a single ecological gradient at a time.

Our method of module detection can point to groups of species among which non-neutral interactions are necessarily at work. However, it does not clearly point to the nature of non-neutral processes at work among modules, and we showed that many distinct ecological scenarios can lead to detecting modules (figure 1). The nature and hypothesized effects of the target ecological gradient and the links between modules and species traits can both contribute to interpreting modules in terms of non-neutral processes, though. In the tropical tree dataset, we retrieved the main conclusions of [15]: a strong effect of the annual rainfall gradient across plots segregated two non-neutral modules associated with deciduous and evergreen species. This pattern is consistent with the expected effects of environmental filtering induced by a gradient of hydric stress. Regarding our analysis of Mediterranean herbaceous communities, we found two species modules that both showed a mild, non-significant decrease in presence probability close to thyme. One module contained competitive grass from the Poaceae family, including one species of the genus *Bromus* (*B. hordeaceus*). We have already mentioned that another species of the same genus (*B. erectus*) has been identified as repelled by thyme in previous studies [31,37], hence potentially inducing a competition release effect on the rest of the plant community. Interestingly, although it was present in the sites considered here, *B. erectus* itself did not appear in the module because it succeeded in being dominant everywhere (see Saint-Martin-de-Londres study details in [22]), including close to thyme, and thus did not switch rank in presence probability with any other species. The other module was more novel and calls for further investigation. Contrary to the tropical forest example and contrary to our expectations, the modules detected in herbaceous communities did not show opposite trends with respect to the gradient of interest (thyme presence) but still switched ranks along the gradient, probably because the presence probabilities of module 1 drop faster than that of module 2, hence indicating a change in fitness ratios. Comparing our two real case studies hence brought a concrete illustration of the fact that rank consistency and spatial covariance of occurrences are well-distinct features.

Identifying the rank consistency of species as a robust and general prediction of neutral theory allowed building a method to detect non-neutral modules that does not assume the existence of an ‘effective number of migrant’ [50], i.e. the assumption that the immigration rate should be proportional to the local *per capita* birth rate at all times. This is an important breakthrough, for this assumption lies at the core of most neutral models to date, even those that relax important instrumental assumptions like the constant community size [19]. The existence of an effective number of migrants is a strong assumption that may rarely hold in nature and apply only to specific systems where adults would be observed, dispersal would occur at the juvenile stage, and all the juveniles within a site (whether local or immigrating) would be regulated in the same way by density dependence during maturation. This may only hold for some plants, corals or other sessile organisms. Even in the latter cases, this may come with serious limitations as it does not allow, for instance, the fertility of local individuals to depend on community density dependence, which can readily arise in entomogamous plant communities due to competition for local pollinators and pollen limitation [51]. Here, we overcame these limits by focusing on the rank consistency of species pairs. Ho­wever, rank consistency is probably not the only pattern worth considering from this perspective, and we expect that other robust predictions of the neutral theory concerning, for example, triplets of species could be derived in the same way, hence further increasing the potential of our approach.

## Data Availability

Our work uses two datasets published elsewhere. The tropical tree dataset used here is available online in the supplementary material ecy2977-sup-0002-DataS1.zip of Laroche *et al*. [15]. The herbaceous community dataset is directly available online [32]. All the code and information needed to reproduce the analyses are provided on a public online repository [22]. Electronic supplementary material is available online [52].
